# Evaluation of acquisition modes for semi‐quantitative analysis by targeted and untargeted mass spectrometry

**DOI:** 10.1002/rcm.9308

**Published:** 2022-05-03

**Authors:** Hannah M. Britt, Tristan Cragnolini, Suniya Khatun, Abubakar Hatimy, Juliette James, Nathanael Page, Jonathan P. Williams, Christopher Hughes, Richard Denny, Konstantinos Thalassinos, Johannes P. C. Vissers

**Affiliations:** ^1^ Institute of Structural and Molecular Biology, Division of Biosciences University College London London UK; ^2^ Institute of Structural and Molecular Biology, Birkbeck College University of London London UK; ^3^ LGC Group Teddington UK; ^4^ Waters Corporation Wilmslow UK

## Abstract

**Rationale:**

Analyte quantitation by mass spectrometry underpins a diverse range of scientific endeavors. The fast‐growing field of mass spectrometer development has resulted in several targeted and untargeted acquisition modes suitable for these applications. By characterizing the acquisition methods available on an ion mobility (IM)‐enabled orthogonal acceleration time‐of‐flight (oa‐ToF) instrument, the optimum modes for analyte semi‐quantitation can be deduced.

**Methods:**

Serial dilutions of commercial metabolite, peptide, or cross‐linked peptide analytes were prepared in matrices of human urine or *Escherichia coli* digest. Each analyte dilution was introduced into an IM separation‐enabled oa‐ToF mass spectrometer by reversed‐phase liquid chromatography and electrospray ionization. Data were acquired for each sample in duplicate using nine different acquisition modes, including four IM‐enabled acquisitions modes, available on the mass spectrometer.

**Results:**

Five (metabolite) or seven (peptide/cross‐linked peptide) point calibration curves were prepared for analytes across each of the acquisition modes. A nonlinear response was observed at high concentrations for some modes, attributed to saturation effects. Two correction methods, one MS1 isotope‐correction and one MS2 ion intensity‐correction, were applied to address this observation, resulting in an up to twofold increase in dynamic range. By averaging the semi‐quantitative results across analyte classes, two parameters, linear dynamic range (LDR) and lower limit of quantification (LLOQ), were determined to evaluate each mode.

**Conclusion:**

A comparison of the acquisition modes revealed that data‐independent acquisition and parallel reaction monitoring methods are most robust for semi‐quantitation when considering achievable LDR and LLOQ. IM‐enabled modes exhibited sensitivity increases, but a simultaneous reduction in dynamic range required correction methods to recover. These findings will assist users in identifying the optimum acquisition mode for their analyte quantitation needs, supporting a diverse range of applications and providing guidance for future acquisition mode developments.

## 1 INTRODUCTION

Mass spectrometry (MS) is a powerful technique, which has developed to the point of being near‐indispensable in several scientific environments. Every day MS is contributing to fundamental research in understanding the molecules of life, from metabolites and lipids, through to proteins and their complexes.^1^ Furthermore, MS plays a vital role in a diverse range of industrial workflows, including quality control, drug detection, and clinical biomarker analysis.^2^, ^3^ It is the depth and breadth of these applications which make MS such a vital analytical tool. For several decades, qualitative MS analysis has been the key strand that cultivated these endeavors, because of the unique ability of MS to identify and characterize a wide array of molecules. MS is able to complete these discovery tasks with a high level of consistency, with an ever‐growing number of technical improvements. In addition to the qualitative benefits of MS, there are now many workflows that focus not only on molecular characterization but also on quantitation.^4^ Quantitative MS is able to measure the amount of each molecule in a sample and has therefore found favor across applications such as proteomics and pharmaceutical analysis.^5^, ^6^ However, quantitative high‐resolution MS is still challenging to perform, not least because of the numerous technical considerations that must be considered.

One technical challenge that must be considered when performing a quantitative high‐resolution MS workflow is selection of the quantitation method. Quantitative MS approaches can be broadly packaged into two distinct families, the first of which is termed the labeling approaches. This set of techniques involves quantitation using popular isotopic species, which comprises techniques such as tandem mass tags (TMT), stable isotope labeling by amino acids (SILAC), and isotopically labeled standards.^7^, ^8^, ^9^, ^10^, ^11^ This suite of quantitation methods has been developed to the point that robust and reproducible workflows exist, and quantitation using these methods is generally considered to be highly accurate when performed correctly. There are, however, also limitations to the labeling techniques: they are expensive, can suffer from incomplete labeling efficiencies, and are limited in the number of samples that can be analyzed in parallel within a single experiment. These challenges in quantitation using labeling approaches have given rise to the second family of techniques, termed label‐free quantitation.^12^, ^13^, ^14^ In the case of the label‐free methods no isotopic labels are required; instead the amount of a given analyte is determined by either chromatographic peak area or spectral counting. Compared to labeling approaches, label‐free quantitation generally requires more straightforward sample preparation and can perform comparative analysis of a greater number of samples within a single experiment. However, given the reliance on peak area or spectral counting within label‐free quantitation workflows, considerable care is required in selecting the sample preparation methodology and instrumental conditions for analysis.

**FIGURE 1:**
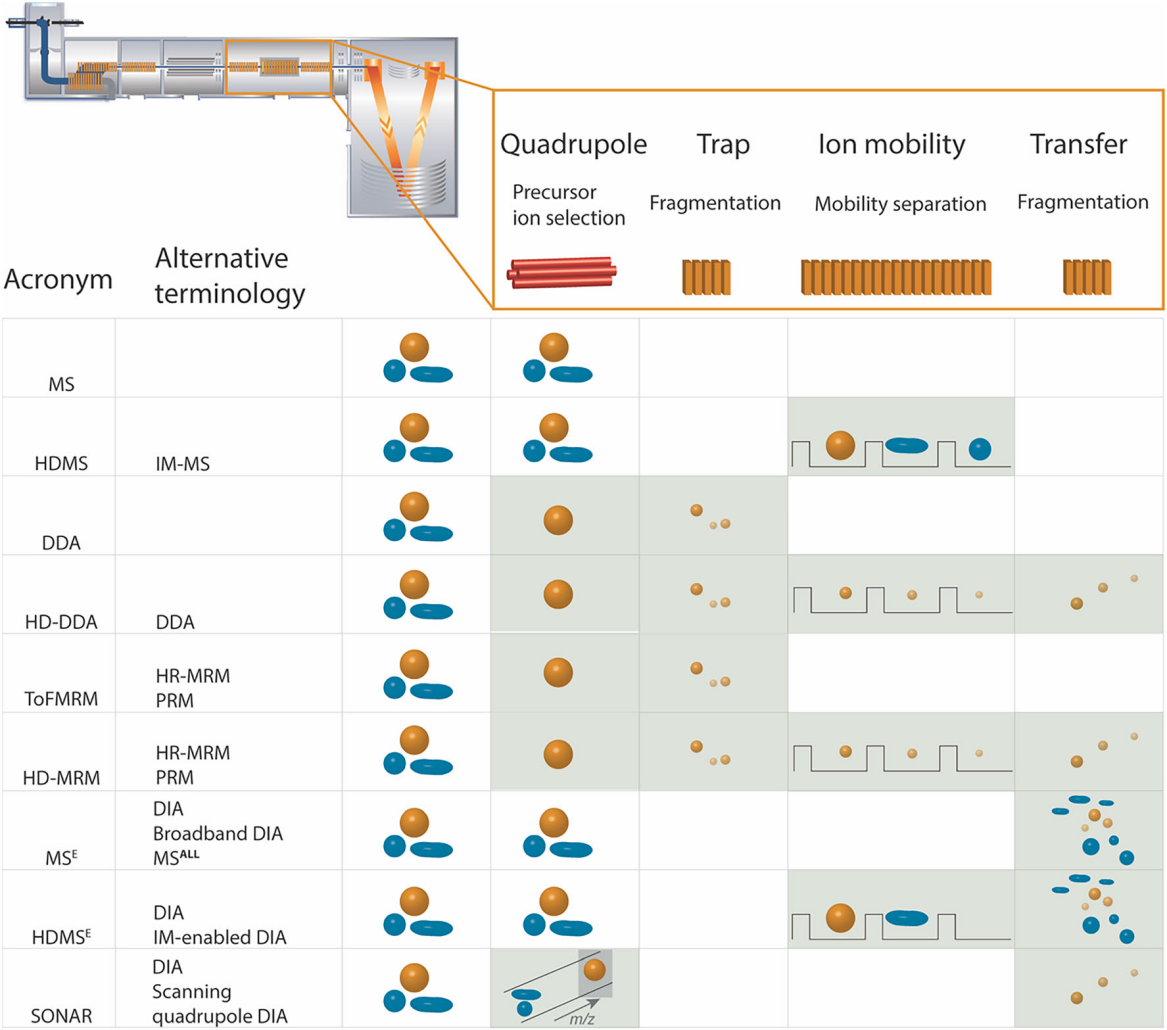
Schematic showing the processes taking place within each portion of the mass spectrometer for acquisition modes available on Synapt G2‐S*i* mass spectrometer. The functions in each region are as follows: (i) the quadrupole can be used in three ways, allowing all ions to pass through (MS, HDMS, MS^E^, HDMS^E^), performing precursor ion selection based on intensity (DDA, HDDDA) or a predefined *m/z* (ToFMRM, HDMRM), or as a scanning quadrupole (SONAR); (ii) CID fragmentation is applied in the trap for a subset of the acquisition modes (DDA, HDDDA, ToFMRM, HDMRM); (iii) for IM‐enabled modes mobility separation is performed next either on precursor ions (HDMS, HDMS^E^) or CID fragments (HDDDA, HDMRM); (iv) CID fragmentation can be performed in the transfer as an alternative to the trap; this is the case for the high energy MS2 experiment that makes up part of the MS^E^ and HDMS^E^ acquisition modes. The outcome of these steps is that PRM acquisition methods (TofMRM and HDMRM) provide MS2 data only, and MS modes (MS and HDMS) MS1 level data. Broadband DIA modes (MS^E^ and HDMS^E^), scanning quadrupole DIA (SONAR), and DDA provide both MS1 and MS2 data. Precursor/product ion color (orange/blue) denotes the ion *m/z*; and the precursor/product ion shape denotes collision cross section (CCS) [Color figure can be viewed at wileyonlinelibrary.com]

Selection of a suitable acquisition mode is one important instrument consideration required for successful label‐free quantitation.^15^, ^16^ The importance of this requirement has been highlighted for several analyte classes by a number of excellent review articles.^4^, ^5^, ^6^, ^17^ The fast expanding field of mass spectrometer development has resulted in the existence of several such acquisition modes, each with their own set of strengths and weaknesses. These acquisition modes include both targeted methods, in which species of interest are predefined based on previous MS characterization, and untargeted acquisition modes, in which species are not defined in advance. Recent work has evaluated the qualitative and quantitative performance of these acquisition methods for the analysis of veterinary drug reference substances and histone posttranslational modifications on Orbitrap instrumentation.^18^, ^19^ The approach presented here parallels this recent work by evaluating semi‐quantitative performance of acquisition modes on an alternative popular class of instrumentation, an IM‐enabled quadrupole orthogonal acceleration time‐of‐flight (oa‐ToF) mass spectrometer. Nine distinct acquisition modes available on this instrument, illustrated in Figure 1, were compared. These acquisition modes include the following: (a) a screening MS1‐only method (MS); (b) the targeted parallel reaction monitoring (PRM) mode known as ToFMRM; (c) an untargeted method for data‐dependent acquisition (DDA); and (d) two untargeted data‐independent acquisition (DIA) methods, one being MS^E^, a broadband DIA method, and the other SONAR, a scanning quadrupole DIA mode. Furthermore, as the oa‐ToF mass spectrometer used in this study contains an IM cell, these acquisition modes (with the exception of SONAR) were also assessed with IM enabled. These modes are denoted using HD in their acronym, known as HDMS, HDMRM, HDDDA, and HDMS^E^, respectively. Ion mobility‐mass spectrometry (IM‐MS) has previously been successful in improving the qualitative analysis of several analyte classes, including small molecules, tryptic peptides, protein complexes, and, for a handful of cases, on cross‐linked peptides.^20^, ^21^, ^22^, ^23^, ^24^, ^25^ For readers requiring a more detailed description of the principles of each acquisition method, this information is provided in Supplementary Note 1. By evaluating these acquisition methods for two common analyte classes, metabolites and peptides, this study will support readers in selection of the optimum acquisition mode for their own label‐free semi‐quantitative analysis. Furthermore, a small‐scale pilot analysis of cross‐linked peptides using the most favorable acquisition modes for semi‐quantitation suggests that the findings would also be applicable to more challenging analyte classes. Given the broad interest in quantitative and semi‐quantitative analysis across the field of MS, these findings would be applicable to a range of users, from those in fundamental research through to clinical and other industrial applications.

## 2 METHODS

### 2.1 Metabolite sample preparation

The following eight metabolite standards were purchased and prepared at a concentration of 100 ng/μL in methanol: AKB‐48 Apinaca 5‐Hydroxypentyl metabolite, AKB‐48 Apinaca 5‐Hydroxy‐pentyl metabolite‐D_4_, JWH‐073 3‐Hydroxybutyl metabolite, JWH‐073 3‐Hydroxybutyl metabolite‐D_5_ (indole‐D_5_), JWH‐250 4‐Hydroxypentyl metabolite, JWH‐250 4‐Hydroxypentyl metabolite‐D_5_, JWH‐122 4‐Hydroxypentyl metabolite, and JWH‐122 4‐Hydroxypentyl metabolite‐D_5_ (all Sigma‐Aldrich, St. Louis, MO, USA). The eight metabolites were combined in equal amounts to form a single stock sample referred to as the metabolite mixture. The mixture was then diluted into pooled human urine obtained from multiple normal donors (Innovative Research, Novi, MI, USA) with the final metabolite concentrations equaling 1, 10, 100, and 1000 ppb in urine, respectively.

### 2.2 Peptide sample preparation

The peptide mixture used for analysis was MassPREP Protein Digestion Standard Mix 1 (Waters Corporation, Wilmslow, UK), consisting of tryptic digested yeast enolase, glycogen phosphorylase B, yeast alcohol dehydrogenase, and bovine serum albumin (BSA). This mixture was diluted into a constant background of 100 ng/μL of MassPREP *Escherichia coli* digest standard (Waters Corporation). This resulted in seven serial dilutions of the peptide mixture at concentrations of 1 amol/μL, 10 amol/μL, 100 amol/μL, 1 fmol/μL, 10 fmol/μL, 100 fmol/μL, and 1 pmol/μL.

### 2.3 Cross‐linked peptide sample preparation

Cross‐linking reactions were conducted as described previously.^24^ In brief, 0.3 mg/mL BSA (Sigma‐Aldrich) and 1 mg bis (sulfosuccinimidyl)suberate (BS3) d0/d12 (Creative Molecule Incorporated) were prepared in 20 mM HEPES at pH 7.6. The cross‐linker was added to the protein and diluted to a final concentration of 2.5 mM BS3 d0/d12. The sample was then incubated at room temperature for 40 min under mild agitation. After incubation, the reaction was quenched by adding 1 M ammonium bicarbonate to a final concentration of 50 mM. The samples were then evaporated to dryness and resuspended in 8 M urea at 1.1 mg/mL concentration. 1% RapiGest (Waters Corporation) was added to a final concentration of 0.1% and incubated with 10 mM dithiothreitol (DTT) at 37°C for 30 min. After incubation, the sample was cooled to room temperature. Iodoacetamine (IAA) was added to a final concentration of 20 mM and the sample incubated in the dark at room temperature for 30 min. The sample was then diluted with 50 mM ammonium bicarbonate to reduce the final concentration of urea to <1 M. Trypsin, 50:1 protein to enzyme (w/w), was added to the sample and the reaction incubated overnight at 37°C with mild agitation. After overnight incubation, enzymatic activity was quenched by adding formic acid to a final concentration of 2% (v/v). The sample was fractionated using Sep‐Pak SPE cartridges (Waters Corporation) to maximize the number of cross‐linked peptides relative to linear peptides, and evaporated to dryness. The cross‐linked BSA (XL‐BSA) sample was reconstituted and diluted into a constant background of 100 ng/μL of MassPREP *E. coli* digest standard (Waters Corporation), which resulted in seven serial dilutions, based on the original BSA concentration attributed as 1 amol/μL, 10 amol/μL, 100 amol/μL, 1 fmol/μL, 10 fmol/μL, 100 fmol/μL, and 1 pmol/μL.

### 2.4 Liquid chromatography

For metabolite analysis, an I‐class LC system (Waters Corporation) was equipped with an HSS T3 1.8 μm 2.1 × 100 mm column (Waters Corporation) operated at 400 μL/min. The gradient was held at 1% B for 0.3 min, followed by a 1%‐50% increase in B from 0.3 to 7 min, and another step from 50% to 70% B in 1 min. Next, the solvent strength was increased to 99% B in 0.1 min, which was held for 1 min, and the column reconditioned for 1 min at initial gradient conditions. Mobile phase A was 0.1% formic acid in water and mobile phase B 0.1% formic acid in acetonitrile. The column temperature was maintained at 45°C and the samples at 8°C. The injection volume equaled 5 μL.

All peptide separations, both linear and cross‐linked, were conducted with a 1.7 μm CSH 130 C18 300 μm × 100 mm column (Waters Corporation) operated at 7 μL/min using an M‐class LC system (Waters Corporation). Here, the gradient was held first at 1% B for 2 min, followed by a 1%‐30% increase from 2 to 30 min, which was held for 2 min. Next, the solvent strength was increased to 85% B in 1 min, which was held for another 2 min, followed by decreasing the solvent strength in 1 min and reconditioning of the column for 23 min at initial gradient concentration. Mobile phase A was 0.1% formic acid in water and mobile phase B 0.1% formic acid in acetonitrile. The column temperature was maintained at 55°C and the samples at 12°C. The injection volume equaled 4.5 μL.

### 2.5 Mass spectrometry

All MS experiments were conducted in duplicate on an IM‐enabled Synapt G2‐S*i* hybrid quadrupole oa‐ToF mass spectrometer (Waters Corporation). The oa‐ToF analyzer was externally calibrated from *m/z* 50 to 1570 using fragment ion data from [Glu]‐Fibrinopeptide B. For CCS calculation, LC‐MS QC Reference Standard (Waters Corporation) was added to each standard, prepared according to instructions provided by the manufacturer.^26^ For metabolite experiments, the capillary voltage was 1.0 kV, sampling cone 25 V, source offset 30 V, source temperature 100°C, desolvation temperature 600°C, cone gas 50 L/h, desolvation gas 1000 L/h, and nebulizer pressure 6 bar. For peptide and cross‐linked peptide experiments, the capillary voltage was 2.3 kV, sampling cone 30 V, source offset 30 V, source temperature 100°C, desolvation temperature 250°C, cone gas disabled, desolvation gas 500 L/h, and nebulizer pressure 6 bar. Further detailed experimental MS conditions and acquisition parameters are summarized in Tables S1 and S2 (supporting information).

For the IM‐enabled acquisition methods, the Trap and Transfer T‐Waves were pressurized with 2 mL/min of Ar. Gas‐phase optimization for the separation of the analytes made use of N_2_. The He gate contained within the IM region was pressurized with 180 mL/min. The IM T‐Wave was pressurized with 90 mL/min, the IM wave velocity was ramped, as described in Tables S1 and S2 (supporting information), and the pulse height held at 40 V during acquisition. Parameters used for extracting data in the IM domain are detailed in Table S3 (supporting information).

### 2.6 Data processing

**FIGURE 2:**
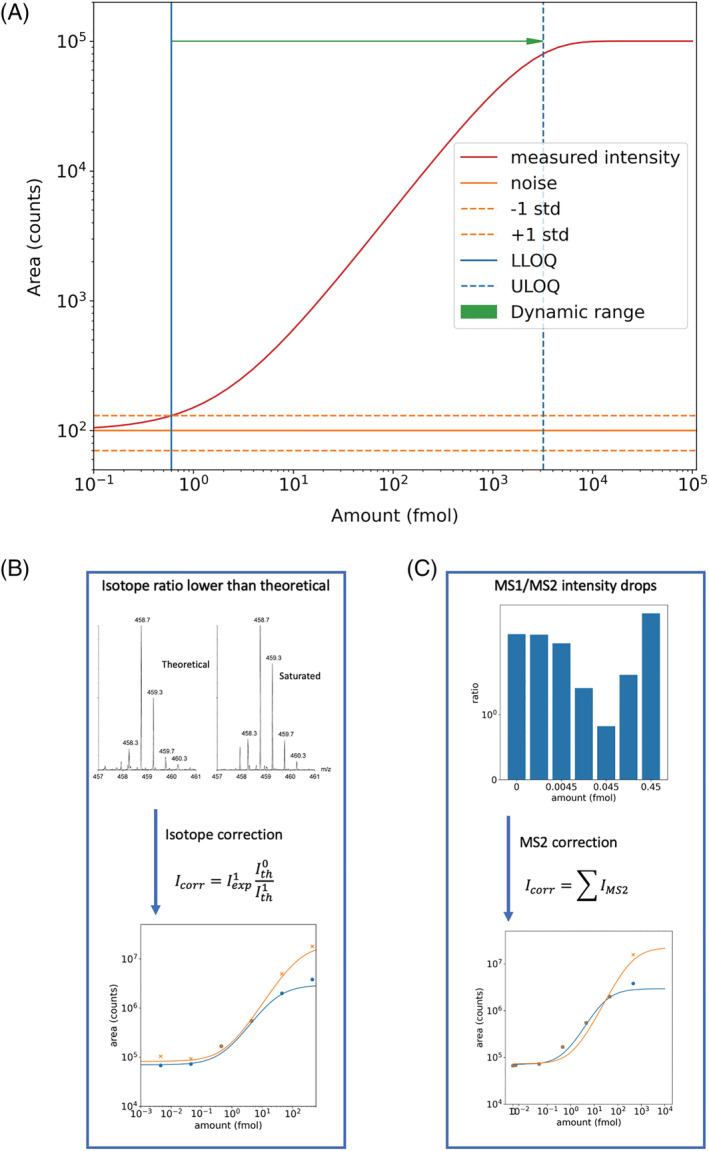
(A) The model used to characterize the calibration curves obtained from analyte quantitation in this study. A sigmoidal shape (red) is fitted to the data considering the effect of noise dominating over signal at low concentrations (orange), and detector saturation at high concentrations. Based on this fit it is possible to define the parameters of interest, LLOQ (solid blue line), ULOQ (dashed blue line), and LDR (green arrow). (B) and (C) show the principles of the isotope and MS2 correction methods, respectively, applied to combat saturation effects at high concentrations. The isotope correct method (B) recognizes a difference in the observed and theoretical ratios of isotope peaks, and a corrected intensity (Icorr) is determined by scaling the experimental value of the first isotope (I^1^exp) by the ratio of the theoretical monoisotopic peak (I^0^th) and first isotope (I^1^th). The result of this approach for one example peptide (NLAENISR) is compared for corrected (orange) vs. uncorrected (blue) data at the bottom of the panel. Equivalent data for the MS2 correction method are shown in (C). In this case, saturation is detected through an observed drop in the MS1/MS2 intensity ratio, and the Icorr is calculated using the sum of the MS2 peak intensities. In the case of both corrections, the effect is clear on the higher concentration scale where saturation effects occur, and the uncorrected and corrected curves diverge [Color figure can be viewed at wileyonlinelibrary.com]

Peak detection was carried out in Skyline and the multidimensional peak detected data exported as transition tables for down‐stream analysis.^27^, ^28^, ^29^ Explicit retention times were specified to aid peak detection and validate the integration. Match tolerances were ±0.1 min and ±0.2 min for the metabolite and (XL) peptide data sets, respectively. A resolution of 20 000 FWHM dimension was specified, from which tolerances are inferred that equal twice the expected peak FWHM in the *m/z* dimension, to extract chromatograms. Observed RMSE mass errors, averaged out over all concentration levels and analytes, equaled 4.1 (MS1 methods) and 3.3 (MS2 method), 2.8 (MS1 methods) and 2.6 (MS2 methods), and 6.4 (MS2 methods) ppm, respectively, for the metabolite, peptide, and XL peptides data sets. Additional analysis was carried out using custom Python 3 scripts, available at https://github.com/ThalassinosLab/Quantitative_fit_MS. To correctly describe whether signal is proportional to the concentration of analyte, modeling of the data is required to consider the effects of noise at low concentration and signal saturation at high concentration. Saturation effects for different mass analyzer types are not uncommon and described elsewhere.^30^, ^31^ Briefly, saturation effects are readily recognized by comparing the isotopic distributions of the highest intensity quartile detections with expected theoretical isotopic distributions. Intensity, both low and high, and isotopic (delta) mass shift errors can both be indicative of detection, that is, saturation, anomalies. This is typical in LC‐MS analyses of biological samples where analytes differ greatly in concentration/amount and can be corrected for using various approaches, either post‐acquisition or during the experiment. Here, two post‐acquisition correction methods are applied and evaluated. As shown in Figure 2A, the data are modeled using exponential fits of type y = C(1‐exp(−x/R)) to detect the central linear range, with inflection points where the effects of saturation or noise become important.

Using the model, conceptually, two parameters of importance were determined in linear space:

- Lower limit of quantification (LLOQ) is recovered by finding the point at which the measured analyte intensity is equivalent to the level of background noise and one standard deviation as determined from multiple replicates and samples.
- Linear dynamic range (LDR), highlighted by the green arrow in Figure 2A, is determined as the ratio difference between the estimated LLOQ and the estimated upper limit of quantification (ULOQ). In this case the ULOQ is defined as the inflection point of the measure intensity curve, above which saturation is apparent and signal no longer scales linearly with concentration.

### 2.7 Data correction methods

Following on from data processing, two post‐acquisition data correction methods were evaluated in an attempt to mitigate the known effects of mass analyzer saturation.^30^, ^31^ The correction methods are as follows:

- MS1 isotope correction—at the level where saturation is observed, the abundance of a saturated isotopic peak for a given species is corrected using the abundances of higher isotopes and their theoretical natural distributions.^32^
- MS2 correction—at the level where saturation is observed, the abundance of the saturated MS1 isotopes for a given species is corrected using the abundance of the non‐saturated precursor/product ion relationships after collision‐induced dissociation (CID) fragmentation for a given analyte.^33^, ^34^, ^35^

Principles of the two correction methods are visually detailed in Figures 2B and 2C, respectively.

## 3 RESULTS AND DISCUSSION

### 3.1 Model development and corrections

Analyte mixtures were prepared to reflect two common sample types subjected to semi‐quantitative MS analysis, namely metabolites and peptides. Accepted reversed‐phase separation materials and methods were applied for both analyte classes.^36^, ^37^ The analyte mixtures comprised an eight‐metabolite mixture diluted into urine matrix, and a four‐protein tryptic digest mixture diluted into an *E. coli* digest background. Five (metabolites) or seven (peptide) serial dilutions of these samples were then analyzed in duplicate using each of the nine acquisition modes, detailed in Figure 1. Duplicate injection results were typically well within a 10% error of each other, suggesting sufficient precision between samples at the individual concentration levels; hence, precision limitations are not expected to affect generalization in any subsequent regression analysis. For example, the median MS1 abundance errors, averaged over all modes of acquisition and on‐column levels, were found to equal to 3.7% and 8.3% for the metabolite and peptide standards, respectively. These figures of merit compare favorably with studies where peak area reproducibility values were reported ranging from 20% to 30% for the majority of the detected features within a metabolomics study pool QC sample (*n* = 17) and ranging from 5% to 15% for peptide standards spiked into a biological matrix (*n* = 5) using DIA methods of acquisition.^37^, ^38^ Example raw MS and MS/MS data for the different acquisition modes are shown in Figure S1 (supporting information), where the ability to conduct MS1, MS2, or combined semi‐quantitation for each mode is also illustrated. Based on these data, multi‐point calibration curves were created for each individual analyte for each of the nine acquisition modes, using MS1, MS2, and combined MS1/MS2 semi‐quantitation as relevant to the particular mode. It was determined from these serial dilution data that the calibration curve features were consistent across analytes and could therefore be modeled with the fit shown in Figure 2A. This model of the data also allowed two parameters, LLOQ and LDR, to be defined, providing metrics for assessing performance of the acquisition modes.

Interpretation of the serial dilution data and the semi‐quantitative results revealed nonlinear response at high sample concentration in the case of some acquisition modes and analytes, attributed to saturation. To mitigate these effects and improve quantitative readout of the underlying data, two correction methods were applied. It was expected that these corrections would increase linearity at higher sample concentrations, extending LDR for quantitation. The first correction, an isotope correction method, corrects the intensity of a saturated monoisotopic peak using the abundances of higher isotopes and their theoretical natural distributions.^32^ The principles of this correction are detailed in Figure 2B. When considered for the example doubly charged peptide NLAENSIR, data shown in the bottom panel of Figure 2B, the isotope correction method proved successful in increasing linear response about twofold. This increased linearity of the data at high concentration results in an improvement in the upper limit of quantitation (ULOQ) and thus LDR. The alternative correction method applied was an MS2‐based correction, shown in Figure 2C, in which the abundance of the saturated species is corrected using the abundance of the non‐saturated precursor/product ion relationships after CID fragmentation for a given analyte.^33^, ^34^, ^35^ This MS2 correction method improves linear response and LDR for peptide NLAENSIR to a similar but slightly smaller order of magnitude compared to the isotope correction method, as shown in bottom panels of Figure 2C. Analogous improvements are observed for metabolites, highlighted in Figure S2 (supporting information), with application of isotope and MS2 corrections to AKB‐48 Apinaca 5‐Hydroxypenytl metabolite showing a 7.1% and 8.4% LDR increase, respectively, compared to uncorrected data.

Dynamic range improvements obtained through applying these correction methods are particularly striking for the IM‐enabled acquisition modes, shown in Table 1 (also depicted visually in Figure S3 [supporting information]). Averaging across both analyte types the net gain in LDR for these modes is equal to ~43%, largely reversing the intrinsic LDR reduction caused by concentration of a continuous ion beam into gated ion packets as part of IM acquisitions.^39^ As a consequence, LLOQ and LDR values across all acquisition modes become far more comparable as a result of these corrections. Ultimately, an overview of averaged uncorrected and corrected values for LLOQ and LDR across acquisition modes, summarized in Table 1, suggests that both correction methods are successful in mitigating signal saturation and increasing LDR. It should, however, be noted that the optimum correction approach varied between analytes; therefore, rather than establishing a universal correction method, users should consider which is more appropriate based on their individual sample needs.

**TABLE 1:** The semi‐quantitative uncorrected and corrected summary figures of merit (average values summed over all analytes with acquisition, integration, and/or computational outliers excluded from the analysis when passing a modified [Iglewicz and Hoaglin] z‐score threshold; errors represent difference in analyte response/ionization efficiency) for all acquisition methods are presented in the following tables for each analyte type

Metabolites (8 metabolites; 5 concentration levels; duplicate injections)						
Acquisition mode	Uncorrected		Isotope corrected		MS2 corrected	
	LLOQ (ppb)	# orders LDR	LLOQ (ppb)	# orders LDR	LLOQ (ppb)	# orders LDR
MS	0.8 ± 0.4	3.0 ± 0.2	0.8 ± 0.4	3.0 ± 0.2	‐‐	‐‐
HDMS	1.4 ± 0.7	2.3 ± 0.1	1.0 ± 0.6	2.6 ± 0.2	‐‐	‐‐
DDA	4.2 ± 3.7	2.6 ± 0.6	4.2 ± 3.7	2.6 ± 0.6	‐‐	‐‐
HDDDA	9.3 ± 5.9	2.1 ± 0.3	12.9 ± 10.9	1.9 ± 0.5	‐‐	‐‐
TofMRM	0.8 ± 0.4	3.0 ± 0.1	‐‐	‐‐	‐‐	‐‐
HDMRM	3.5 ± 2.3	2.2 ± 0.5	‐‐	‐‐	‐‐	‐‐
MS^E^	0.8 ± 0.5	3.0 ± 0.3	0.8 ± 0.5	3.1 ± 0.3	0.7 ± 0.3	3.0 ± 0.2
HDMS^E^	1.3 ± 0.8	2.3 ± 0.1	1.0 ± 0.6	2.7 ± 0.3	1.0 ± 0.5	2.6 ± 0.2
SONAR	0.6 ± 0.4	3.1 ± 0.3	0.6 ± 0.5	3.2 ± 0.4	0.6 ± 0.4	3.2 ± 0.3

Peptides (12 peptides; 7 concentration levels; duplicate injections).						
Acquisition mode	Uncorrected		Isotope corrected		MS2 corrected	
	LLOQ (fmol)	# orders LDR	LLOQ (fmol)	# orders LDR	LLOQ (fmol)	# orders LDR
MS	0.4 ± 0.2	4.0 ± 0.2	0.4 ± 0.2	4.0 ± 0.2	‐‐	‐‐
HDMS	0.5 ± 0.4	2.8 ± 0.7	0.8 ± 1.0	2.9 ± 0.7	‐‐	‐‐
DDA	2.3 ± 2.6	3.2 ± 0.8	1.7 ± 2.2	3.1 ± 0.7	‐‐	‐‐
HDDDA	0.5 ± 0.6	3.8 ± 0.7	0.5 ± 0.6	3.8 ± 0.7	‐‐	‐‐
TofMRM	0.1 ± 0.0	4.0 ± 0.9	‐‐	‐‐	‐‐	‐‐
HDMRM	40.9 ± 48.7	1.7 ± 0.8	‐‐	‐‐	‐‐	‐‐
MS^E^	3.3 ± 3.6	3.2 ± 0.7	3.7 ± 3.7	3.2 ± 0.6	3.3 ± 3.5	3.2 ± 0.7
HDMS^E^	1.0 ± 1.0	2.7 ± 0.6	1.6 ± 1.9	2.7 ± 0.6	1.2 ± 1.1	3.2 ± 0.6
SONAR	3.1 ± 3.0	3.2 ± 0.5	4.1 ± 3.8	3.2 ± 0.5	3.1 ± 3.0	3.3 ± 0.5

Cross‐linked peptides (3 cross‐linked peptides; 7 concentration levels; duplicate injections).		
Acquisition mode	LLOQ (fmol)	# orders LDR
ToFMRM	1.2 ± 0.7	3.5 ± 0.5
MS^E^	12.2 ± 13.9 (4.2 ± 0.6)*	2.6 ± 0.5 (2.9 ± 0.1)*
HDMS^E^	5.0 ± 1.9	2.9 ± 0.1

*Note*. Semi‐quantitation of metabolites and peptides determined at MS1 level for MS, HDMS, DDA, HDDDA, SONAR, MS^E^, and HDMS^E^, whereas ToFMRM and HDMRM modes are based on MS2 level. For cross‐linked peptides, all semi‐quantitation are performed at MS2 level; ‐, not applicable.

* DTHK[BS3]SEIAHR_FK[BS3]DLGEEHFK excluded.

### 3.2 Evaluation of acquisition modes

To evaluate the suitability of acquisition modes for semi‐quantitative MS analysis of metabolites and peptides, readouts for each acquisition mode were averaged over each analyte class. Averaging over a number of analytes of a given class in this way, each of which will have a unique LLOQ and LDR, allows comparison of average results for each acquisition mode. This approach is considered more appropriate for assessing semi‐quantitation in terms of the multicomponent mixtures discussed in this manuscript, rather than basing performance findings on the results of an individual analyte. The average LLOQ and LDR figures obtained for each analyte class using this approach are presented for each acquisition mode in Table 1. The presented data consider semi‐quantitation at the levels appropriate for the relevant applied acquisition method. Although absolute LLOQ and LDR values for each acquisition mode are a function of analyte type, similar sigmoidal distributions (peptide example shown in Figure S4 [supporting information]) are observed for both peptide and metabolite analytes. As a result, the conditions and observations on the performance of each acquisition mode can be generalized for both analyte classes, allowing the semi‐quantitative response of each method to be compared. Viewing acquisition mode performance in this way, agnostic to a specific analyte class, helps to make the findings more broadly applicable to a more diverse range of semi‐quantitative MS applications.

Review of the results from the DDA and IM‐assisted DDA (HDDDA) acquisition modes found a higher level of variation between replicate samples compared to the other acquisition methods, particularly in terms of abundance, as shown by the results summarized in Table 1. This variation was especially pronounced at MS2 level, such that metrics of interest were either not calculated or are considered unreliable. Furthermore, DDA methods underperformed from a semi‐quantitative perspective in terms of both sensitivity and LDR where it was possible to calculate metrics at MS1 level. These observations serve to support the known challenges associated with the use of DDA for quantitation and suggest it may be better suited to qualitative or discovery‐type experiments. These challenges associated with label‐free‐based MS2 quantitation using DDA acquisitions are discussed in greater detail elsewhere.^15^, ^19^

At the opposite end of the semi‐quantitative performance scale, as shown in Table 1, are PRM variant ToFMRM, DIA methods MS^E^ and SONAR, and the MS screening method. For each of these latter methods, MS1 semi‐quantitation generally afforded analysis over a larger LDR than MS2 semi‐quantitation, with the effects being most noticeable for the peptide analytes because of accessing a wider concentration range. Despite performing well in terms of LDR and LLOQ, the MS acquisition mode is an MS1 only method, limiting the characterization information that it can provide. As such, additional care in analyte identification and detection is required, especially at low concentrations where mass accuracy is reduced. Given this, although MS analysis may be suitable for certain targeted experiments, it is not considered a practical method for analysis of multicomponent samples, which can be low abundant and contain a complex matrix similar in nature to the target analytes. In a similar way, some care may be required in the use of the scanning quadrupole DIA mode SONAR for semi‐quantitative MS analysis based on practicalities of use. Although this acquisition mode performs well, across all semi‐quantitative metrics especially for the metabolite standards, it has a reduced duty cycle compared to other DIA methods, which means that it practically can be less suited for semi‐quantitative analysis. The two remaining high‐performing non‐mobility acquisition modes are ToFMRM, and MS^E^. The two modes are largely comparable across LLOQ, LDR, and signal‐to‐noise ratio, with a preference for the ToFMRM acquisition mode, especially for peptides, in terms of LLOQ and LDR metrics. As a result, this evaluation suggests that either mode would be suitable for the semi‐quantitative analysis of complex biological samples. However, it should be noted that the PRM methods like ToFMRM require more knowledge of the system being studied and are more user intensive to set up.^40^ To emphasize this particular aspect, providing further increased sensitivity and dynamic range, two dedicated TofMRM methods, both aimed at improving instrument duty cycle for a selected *m/z* value, a set of values, or range, were applied for the analysis of the peptide samples. These methods, TofMRM_EDC_ and TofMRMsens, are detailed in Supplementary Note 1, and the results are described in Table S4 (supporting information). Moreover, aspects that are typically more associated with multiple reaction monitoring (MRM) analysis, such as interscan delay times and dwell time, affecting duty cycle and thus the number of points per peak, must also be considered.^41^ Interestingly, the semi‐quantification results for these methods suggest that high‐resolution mass analyzers approach LDR and LLOQ levels normally obtained with MRM experiments conducted on tandem quadrupole instrumentation, which is seen as state‐of‐the‐art. The main limitation appears to be the ability to discriminate signal from noise for a given acquisition method.

Comparison of the semi‐quantitative performance of non‐mobility acquisition modes with their IM‐enabled counterparts was also carried out using the LLOQ and LDR metrics shown in Table 1. Unsurprisingly, all IM‐enabled modes exhibited a decreased LDR compared to their non‐mobility counterparts, which can be partially mitigated by application of correction methods as previously discussed. The IM‐enabled HDMS^E^ and HDMRM modes show comparable LLOQ values relative to their non‐mobility equivalents. However, relative values vary between analyte types, attributed to differences in experimental conditions, which highlights that the metrics described are purely estimates to aid in assessing relative performance of the acquisition methods. IM‐enabled modes do show an improved signal‐to‐noise ratio compared to their counterparts, likely because of the reduction in noise afforded by IM separation.

### 3.3 Semi‐quantitative analysis of XL‐BSA

Metabolites and peptides are some of the most common analytes subjected to analysis by quantitative MS; however, they are certainly not the only species for which such analysis would be beneficial. One newer area in which quantitative analysis is of interest is in the field of cross‐linking‐mass spectrometry (XL‐MS), where success has been achieved using both isotopic labeling and label‐free approaches for (semi‐) quantitation to date.^42^, ^43^, ^44^, ^45^, ^46^ Quantitation in the context of XL‐MS facilitates comparative analysis of multiple samples in parallel, characterizing variation in protein structure and interactions under different conditions. It is acknowledged, however, that quantitative XL‐MS is a challenging area of research, due in part to the variation in cross‐linked peptide abundance within a sample, and ion suppression effects that could result from this. As such, it was of interest to discover whether the principles observed for peptides and metabolites discussed so far would also hold within this context.

The test for semi‐quantitation of cross‐linked peptides was performed using a sample of XL‐BSA, and contained both linear and cross‐linked peptides, all of which result from a single protein. It is acknowledged that this is a relatively simple sample compared to many standard XL‐MS experiments; however, given that a multi‐laboratory study has showed large variation in the number of cross‐links observed, it is sufficient to provide proof of principle within this context.^47^ This sample also has the benefit of being previously well characterized on a Synapt platform, where XL‐BSA analysis in isolation at a relatively high concentration, following fractionation, identified cross‐linked peptide precursor and product ions with the best response.^24^ This allowed the subsequent experiments to focus purely on semi‐quantitative ability rather than qualitative aspects of the XL‐MS workflow. Finally, the choice of XL‐BSA as the test sample for semi‐quantitation of cross‐linked peptides provided a high concentration, such that sufficient analyte amount was present to facilitate use of the chromatographic formats described in the preceding paragraphs. Although it is acknowledged that nanoscale LC is normally the preferred choice, affording improved concentration sensitivity for sample limited cases applications, relative LLOQ and LDR, are not affected by column diameter and thus this choice of column format should not prohibit generalization of the observed findings.^48^

**FIGURE 3:**
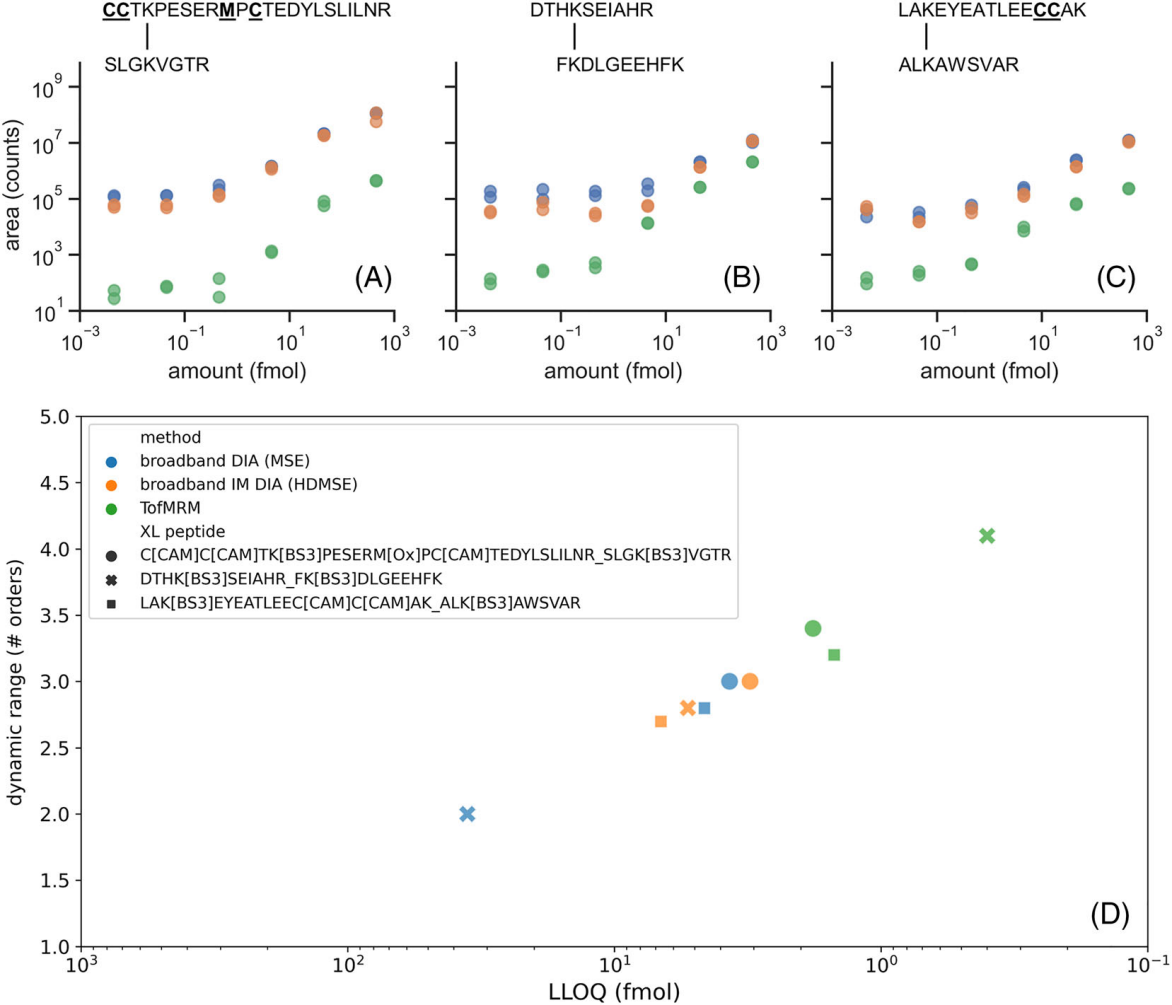
The top panels show quantitative response curves for duplicate injections across three acquisition modes for XL‐BSA peptides: (A) C[CAM]C[CAM]TK [BS3]PESERM[ox]PC[CAM]TEDYLSLILNR_SLGK[BS3]VGTR; (B) DTHK[BS3]SEIAHR_FK[BS3]DLGEEHFK; (C) LAK[BS3]EYEATLEEC[CAM]AK_ALK[BS3]AWSVAR with modified amino acids bold underlined. The three acquisition modes featured are MSE (blue), HDMS^E^ (orange), and ToFMRM (green). The bottom panel (D) presents the LLOQ (inversed log scale) and LDR obtained from the quantitative response curves for each cross‐linked peptide analyzed under each acquisition mode at MS2 level for the three modes of acquisition [Color figure can be viewed at wileyonlinelibrary.com]

Having identified the most optimal performing acquisition modes for metabolite and peptide semi‐quantitation, these were then applied to determine whether their semi‐quantitative abilities would hold for cross‐linked peptides. The three methods chosen, based on both practicalities and performance, were: (a) broadband DIA (MS^E^) mode; (b) IM‐enabled broadband DIA mode (HDMS^E^); and (3) PRM mode (TofMRM). The acquisition methods were applied to analyze serial dilutions of XL‐BSA digest in duplicate within an *E. coli* digest matrix. Based on previous DDA analysis, the three most abundant XL‐BSA peptides were selected within the data to prepare calibration curves.^24^ Concentration‐dependent plots for each peptide (Figures 3A–3C) show that the XL‐BSA peptides appear at low abundance, resulting in plots that are approximately linear across the concentration range tested (mean *R* ^2^ of 0.97). Given the linearity of the data, it was concluded that these cross‐linked peptides do not suffer from saturation effects, even at the highest concentrations analyzed here, and therefore no further correction methods were applied.

LLOQ and LDR metrics obtained at MS2 level, for consistency, for each cross‐linked peptide across the three acquisition modes are shown in Figure 3D. Although MS^E^ and HDMS^E^ theoretically allow MS1 level quantitation in addition to MS2, for the cross‐linked peptides interferences hampered the ability to make use of this effectively. These values are given for each individual cross‐linked peptide, rather than averaging across analytes as done for corresponding metabolite and peptide data. Because of the low number of analytes and stoichiometry‐related aspects associated with cross‐linked peptide applications, averaging LLOQ and LDR values across the cross‐linked peptides was not deemed to be appropriate. Given that the major challenge is low cross‐linked peptide abundance, LLOQ is of key importance in determining suitability of each mode for semi‐quantitation for this application. The ToFMRM method has by far the best LLOQ for all three XL‐BSA peptides compared to the other methods, making it the best performing. LLOQ values for MS^E^ and HDMS^E^ acquisition modes are less favorable compared to ToFMRM, with both methods illustrating similar semi‐quantitative performance metrics. Interestingly, one XL‐BSA peptide showed an improved LLOQ when IM was enabled, suggesting a specificity challenged case that was addressed by IM separation. It should also be noted that these LLOQ values were achieved with an unfractionated sample on a relatively large inner diameter LC‐MS setup; therefore, it is expected that lower detection and quantification limits may be feasible by using other strategies, such as fractionation of peptides and use of nanoscale LC systems.

Despite their reduced abundance compared to linear peptides, cross‐linked peptide concentrations can still span a substantial LDR. As such it is also important to consider the LDR metric when determining the suitability of acquisition modes for the semi‐quantitation of cross‐linked peptides. For example, the ToFMRM LDR varies considerably between XL‐BSA peptides, from the highest at 4.1 orders to the lowest at 3.2. As observed for the LLOQ values, the LDR for the broadband DIA methods MS^E^ and HDMS^E^ is similar but reduced compared to ToFMRM LDR. The peptide showing an LLOQ improvement under IM‐enabled conditions showed a similar improvement in LDR, suggesting that both metrics discussed here can be influenced by IM in a cross‐linked peptide‐dependent manner without an obvious trend observed.

**FIGURE 4:**
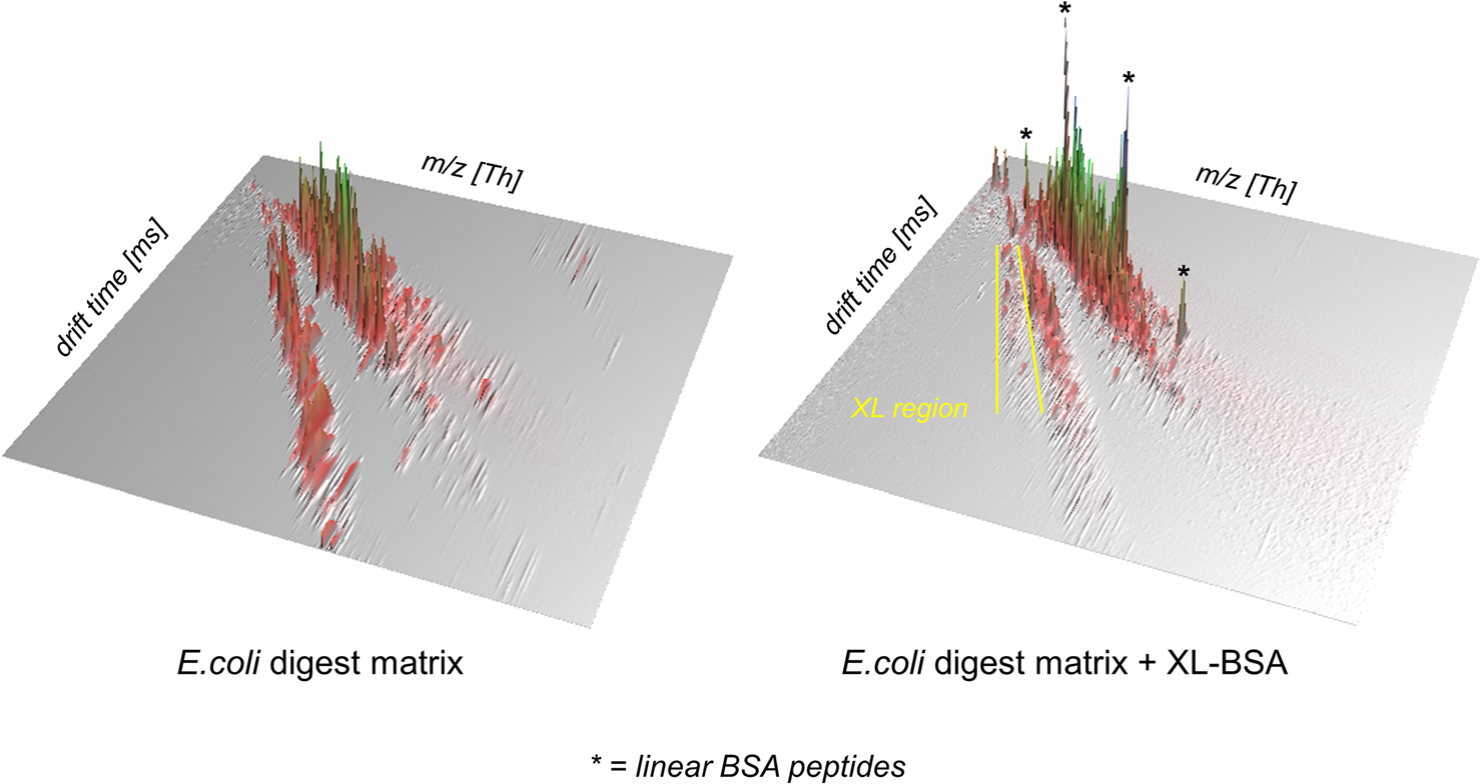
A 3D construction showing HDMS^E^ data in a plot of intensity against drift time and *m/z*. The left panel shows data for the background matrix only, an *Escherichia coli* digest, and the right panel contains data from the matrix plus XL‐BSA digest. The linear BSA peptides in the sample are highlighted by * and come at a comparable drift time to linear matrix peptides. The capability of IM‐MS to isolate higher charge state cross‐linked peptides from linear peptides and background noise is shown by a distinct drift time region containing cross‐linked peptides, shown in yellow on the right‐hand panel [Color figure can be viewed at wileyonlinelibrary.com]

Given the interesting cross‐linked peptide‐dependent effects observed for the HDMS^E^ acquisition mode, the role of IM in cross‐linked peptide semi‐quantitation was further probed. During analysis of XL‐BSA peptides, IM allowed distinct IM separation of cross‐linked peptides from the complex background matrix, including their separation from all linear peptides from BSA and *E. coli* digest. The fact that these higher charge state cross‐linked peptides separate out in the IM domain is consistent with the expectation that they behave similar to other peptide and protein analytes in terms of their IM‐MS charge separation dependency.^39^, ^49^, ^50^, ^51^ As shown in Figure 4, the three XL‐BSA peptides selected for characterization appeared at a distinct drift time distribution compared to all other mixture components, suggesting a fingerprint region for identification of cross‐linked peptides. In addition, this IM separation improves signal‐to‐noise and reduces the susceptibility of XL‐BSA peptides to suppression caused by higher abundance species which elute within the same chromatographic retention window. These observations are consistent with previous observations of IM adding an extra dimension of separation, and the ability of IM to improve analysis of cross‐linked peptides.^25^, ^52^, ^53^ It is also hypothesized that the packaging of a continuous ion beam by IM, problematic at high concentrations, may have a positive effect at the low concentrations of cross‐linked peptides by enhancing detectability.

## 4 CONCLUSION

Label‐free quantitative MS is an important and versatile tool, which supports a large number of research and commercial applications, covering analyte types from small molecules to proteins. Within this study, the available acquisition modes for semi‐quantitation on an IM‐enabled oa‐ToF have been successfully compared, with the aim of assisting users in selecting the most suitable workflow for their applications. Each of the nine available acquisition modes allowed calibration curves to be generated from serial dilutions of metabolite and peptide mixtures. The modeling and evaluation of these calibration data identified the presence of a known quantitation problem, saturation at high analyte concentrations. The two methods presented in this study to address this known issue, isotope correction and MS2 correction, were both found to assist in an up to twofold increase in the LDR for semi‐quantitation. This increase will be important for applications working at the higher concentrations or over a wide concentration range, where users will benefit from applying these corrections. Selecting the correction method should be considered carefully; however, as while both are useful, the optimum correction is analyte specific and will therefore vary by application.

For any given quantitative MS experiment, a number of factors will need to be considered when selecting the acquisition mode for a study, specifically LLOQ and LDR, which were determined for each acquisition mode as part of this work. These values were determined to be agnostic to analyte type; therefore, the findings herein will be applicable to a number of future studies. Based on these metrics, DIA modes such as MS^E^ and SONAR, along with PRM mode ToFMRM, were the best performing for label‐free semi‐quantitation by MS. This is consistent with findings from the wider community, in which these modes are the most commonly selected for quantitative and semi‐quantitative MS. IM‐enabled modes also showed benefits for semi‐quantitative analysis within these framework metrics, particularly at low concentrations, because of their ability to improve the signal‐to‐noise ratio. Although there is some reduction in LDR, which can be repaired by applying the corrections discussed earlier, which may make use of IM‐enabled modes challenging for working at the higher end of the concentration range. In addition to considering these metrics, when selecting an acquisition mode for quantitation, other factors must come into play. For example, instrument capabilities and throughput/speed of analysis may alter which modes can be practically incorporated into a workflow. Furthermore, limited knowledge of the analytes would require the user to select the DIA methods over the PRM workflow.

In addition to evaluating acquisition modes for semi‐quantitation across two popular analyte types, this study applied selected semi‐quantitation modes to more challenging analytes in a proof of principle experiment. This analyte family, cross‐linked peptides, presents additional challenges in label‐free quantitation because of the relatively low analyte abundance within the sample mixture, and their susceptibility to ion suppression as a result. Importantly, both PRM (ToFMRM) and DIA (MS^E^ and HDMS^E^) acquisition methods performed well in their LDR and LLOQ metrics for these challenging analytes, just as they did for the metabolite and peptide analyses. Although ToFMRM performed most favorably, notably by exhibiting the lowest achievable LLOQ, all methods would in principle be suitable for semi‐quantitative analysis, considering the additional factors discussed, such as prior knowledge of the cross‐linked analytes. It is anticipated that this will provide context and aid discussion as the field of XL‐MS continues to look toward suitable acquisition methods for quantitative analysis. IM‐enabled acquisition modes are also being considered by the XL‐MS community, and the results herein support findings from previous studies suggesting that IM can be beneficial to the analysis of cross‐linked peptides.^25^, ^52^, ^53^

5

### PEER REVIEW

The peer review history for this article is available at https://publons.com/publon/10.1002/rcm.9308.

## Supporting information


**FIGURE S1** 1D extracted MS1 (left) and MS2 (right) data for a doubly charged analyte eluting at a retention time of 16.6 min within the peptide sample of tryptic peptides spiked into an *Escherichia coli* matrix. The analyte ion is attributed to the DIVGAVLK peptide of yeast alcohol dehydrogenase. MSE data can additionally be extracted in 2D, whereas HDMSE and SONAR data can be further extracted in 3D
**FIGURE S2** Comparison of uncorrected IM‐enabled DIA (HDMSE) data for analyte AKB‐48 Apinaca 5‐Hydroxypentyl with data corrected using the “isotope” and “MS2” methods. Blue dots = summed precursor signal (isotopes) uncorrected and corrected data points; blue line = sigmoidal fit; blue dashed lines = computational LLOQ and ULOQ estimates; orange line = linear fit
**FIGURE S3** Isotopic correction metabolites averaged out over all amounts injected on‐column for IM‐MS (HDMS) and IM‐enabled DIA (HDMSE) acquisition methods (left: orange = uncorrected; blue = corrected), the average gain in signal for the individual metabolite isotopes (top right: dark blue = IM‐MS [HDMS]; light blue = IM‐enabled DIA [HDMSE]), and the overall signal gain summed over all isotopes and metabolites (bottom right: orange = uncorrected; blue = corrected)
**FIGURE S4** Example corrected (using the best of the two correction methods) and uncorrected calibration curves for all acquisition methods are presented for peptide NLAENISR of rabbit glycogen phosphorylase B. Blue dots = measured response; blue lines = least square linear fit; blue dashed lines = computational LLOQ and ULOQ estimates; orange dashed lines = sigmoidal fit
**TABLE S1** The following MS acquisition parameters were used for metabolites throughout this study; ‐, not applicable
**TABLE S2** The following MS acquisition parameters were used for (cross‐linked) peptides throughout this study (‐, not applicable)
**TABLE S3** The following reference TWCCSN2 values were used for metabolites throughout this study
**TABLE S4** The semi‐quantitative uncorrected figures of merit (average values summed over all peptides with acquisition, integration, and/or computational outliers excluded from the analysis when passing a modified [Iglewicz and Hoaglin] z‐score threshold; errors represent difference in analyte response/ionization efficiency) for TofMRMEDC and TofMRMsens modes of acquisition for peptides. Semi‐quantitation is based on MS2 level
**TABLE S5** The following Skyline data files are available on Panorama (https://panoramaweb.org/Age9Ya.url) public repository

## Data Availability

The data that support the findings of this study are openly available on the Panorama public repository using the URL https://panoramaweb.org/Age9Ya.url.
