# Supplementary material for: Evaluation of acquisition modes for semi‐quantitative analysis by targeted and untargeted mass spectrometry
Source: Rapid Commun Mass Spectrom. 2022 May 3;36(13):e9308. doi: 10.1002/rcm.9308 (PMC9287043; doi:10.1002/rcm.9308)
Supplement: Supplementary file 1 — FIGURE S1 1D extracted MS1 (left) and MS2 (right) data for a doubly charged analyte eluting at a retention time of 16.6 min within the peptide sample of tryptic peptides spiked into an Escherichia coli matrix. The analyte ion is attributed to the DIVGAVLK peptide of yeast alcohol dehydrogenase. MSE data can additionally be extracted in 2D, whereas HDMSE and SONAR data can be further extracted in 3D FIGURE S2 Comparison of uncorrected IM‐enabled DIA (HDMSE) data for analyte AKB‐48 Apinaca 5‐Hydroxypentyl with data corrected using the “isotope” and “MS2” methods. Blue dots = summed precursor signal (isotopes) uncorrected and corrected data points; blue line = sigmoidal fit; blue dashed lines = computational LLOQ and ULOQ estimates; orange line = linear fit FIGURE S3 Isotopic correction metabolites averaged out over all amounts injected on‐column for IM‐MS (HDMS) and IM‐enabled DIA (HDMSE) acquisition methods (left: orange = uncorrected; blue = corrected), the average gain in signal for the individual metabolite isotopes (top right: dark blue = IM‐MS [HDMS]; light blue = IM‐enabled DIA [HDMSE]), and the overall signal gain summed over all isotopes and metabolites (bottom right: orange = uncorrected; blue = corrected) FIGURE S4 Example corrected (using the best of the two correction methods) and uncorrected calibration curves for all acquisition methods are presented for peptide NLAENISR of rabbit glycogen phosphorylase B. Blue dots = measured response; blue lines = least square linear fit; blue dashed lines = computational LLOQ and ULOQ estimates; orange dashed lines = sigmoidal fit TABLE S1 The following MS acquisition parameters were used for metabolites throughout this study; ‐, not applicable TABLE S2 The following MS acquisition parameters were used for (cross‐linked) peptides throughout this study (‐, not applicable) TABLE S3 The following reference TWCCSN2 values were used for metabolites throughout this study TABLE S4 The semi‐quantitative uncorrected figures o [file RCM-36-0-s001.docx]

**Evaluation of Acquisition Modes for Semi-Quantitative Analysis by Targeted and Untargeted Mass Spectrometry**

**Supplementary Information**

Hannah M. Britt^1^‡, Tristan Cragnolini^1,2^‡, Suniya Khatun^1^, Abubakar Hatimy^1^, Juliette James^1^, Nathanael Page^1,3^, Jonathan P. Williams^4^, Christopher Hughes^4^, Richard Denny^4^, Konstantinos Thalassinos^1,2^* and Johannes P.C. Vissers^4^*

1. Institute of Structural and Molecular Biology, Division of Biosciences, University College London, London, WC1E 6BT, United Kingdom
2. Institute of Structural and Molecular Biology, Birkbeck College, University of London, London, WC1E 7HX, United Kingdom
3. LGC Group, Teddington, TW11 0LY, United Kingdom
4. Waters Corporation, Wilmslow, SK9 4AX, United Kingdom

‡ These authors contributed equally to this manuscript.

* Corresponding authors: Konstantinos Thalassinos (k.thalassinos@ucl.ac.uk) and Johannes P.C. Vissers (hans_vissers@waters.com).

Keywords: acquisition modes; quantitation; targeted mass spectrometry; untargeted mass spectrometry; ion mobility.

SupplEmentary NOTE 1

The acquisition modes that have been applied for the semi-quantitative analysis of metabolites and (cross-linked) peptides are graphically summarized in Figure 1. Based on application, they can be grouped into the following categories, *i)* screening (MS and HDMS), *ii)* discovery/non-targeted data dependent acquisition (DDA and HDDDA), *iii)* targeted acquisition (TofMRM, TofMRM_EDC_ and HDMRM), and *iv)* discovery/non-targeted data independent acquisition (MS^E^, HDMS^E^ and SONAR). Categories *ii* and *iii* can be combined from an acquisition perspective and will therefore be discussed as one.

*i) screening*

In MS mode of acquisition, the quadrupole analyzer (Q) operates in wide band RF mode to pass all ions transported from the source via a step-wave ion guide. The Trap and Transfer regions of the traveling wave ion guide (TWIG) cell of the instrument both operate at low Collision Induced Dissociated (CID) energy so that only precursor ion mass information is recorded by the oa-ToF analyzer.

In the instance of IM-MS (HDMS) acquisitions, ions are accumulated in the Trap T-Wave and periodically released into the Ion Mobility (IM) T-Wave, where they separate according to their mobility through action of a continuous train of DC pulses.^1,2^ The ions separated in this way are then propelled through the Transfer T-Wave into the orthogonal acceleration Time-of-Flight (oa-ToF) analyzer for mass analysis and their individual mobility drift times and masses recorded.

*ii) discovery/non-targeted data dependent and iii) targeted acquisition(s)*

This group of acquisition methods have in common that precursor ions are Q mass-selected and undergo CID within either the Ar pressurized Trap or Transfer regions of the instrument to form product ions/spectra. In the instance of Data Dependent Acquisition (DDA) discovery/non-targeted experiments, the MS to MS/MS switch decisions are made during the acquisition based on pre-defined workflow criteria, *e.g.* precursor intensity, charge state, mobility, *etc.*, whereas in the case of targeted methods, *m/z* and retention times, and optionally mobility values, are typically predefined.

DDA experiments were first described in the late 1990’s and many variants proposed, often prompted by advances in technology or instrumentation development, such as parent ion discovery experiment; however, the basic principles have not changed and are typical for the DDA method applied in this study as well, where the instrument was allowed to conduct MS/MS on the 20 most abundant multiply charged precursor ions detected in a survey scan.^3–5^ CID collision energy voltages were calculated and applied on the fly based on precursor *m/z* and charge state using a lookup table strategy. In the current study, two IM enabled variants were applied, aimed at increasing the specificity of the experiment or improving the duty-cycle of the instrument and thereby increasing sensitivity. The survey scan of these methods does not differ from a normal DDA experiment; however, CID fragmentation can be conducted pre or post IM separation, in the Trap or Transfer region, respectively, resulting in either precursor or product ion separation. In the former case, Q co-isolated precursor ions are further separated based on their difference in ion gas-phase mobility, and in the latter case, separation of the product ions is realized, which can provide up to a ten-fold sensitivity increase over specific *m/z* ranges. This is achieved by synchronization of the orthogonal acceleration pusher frequency with the *m/z* ranges of the product ions as they exit the TriWave region of the instrument.^6^ Occasionally, this method is referred to as High Duty Cycle (HDC) DDA.

High-resolution targeted acquisitions, such as parallel reaction monitoring (PRM) experiments, including the TofMRM, TofMRM_EDC_, TofMRM_sens_ and HDMRM methods only differ from DDA experiments in the a *priori* definition of the compounds that must be selected for an MS/MS experiment. The variants applied here are described in great detail elsewhere.^7,8^ Briefly, in normal TofMRM mode, *m/z* values, quadrupole isolation widths, retention times and collision energies are all specified within the method. In the case of Enhanced Duty Cycle (EDC) enabled TofMRM_EDC_ and , TofMRM_sens_ methods, like for the previously mentioned IM enabled DDA HDC method, synchronization of the pusher for selected target *m/z* values/ranges is applied to improve duty cycle. In contrast to the DDA method, however, these targeted *m/z* ranges also must be predefined and typically cover a single or just a few product ions.^7,8^ The HDMRM IM enabled variant is aimed at increasing specificity by applying additional IM separation on possibly quadrupole co-isolated precursor ions and applying CID fragmentation in the transfer region of the T-wave device.

*iv) discovery/non-targeted data independent acquisition*

Data Independent Acquisitions (DIA) do not attempt to select precursor ions for MS2 fragmentation, thereby preventing an intensity-based selection bias, and in terms of available MS2 time, as in the case of DDA acquisitions, duty cycle related limitations. Instead, all ionized peptides of a given sample that fall within a specified mass and/or mobility range are fragmented in a systematic and unbiased manner using either large precursor isolation windows or no isolation windows at all. The DIA methods applied in this study all use an alternate scanning approach, first described for the neutral loss detection and parent ion discovery for phosphopeptide analysis.^5^ Typically, precursor related information is collated in a so-called low energy (MS1) experiment (scan), which is followed by a high-energy (MS2) experiment. This process is repeated during the course of the LC-MS run so that accurate mass precursor and product ion data are collected for all detectable ions.

In an MS^E^ experiment, which would classify as a broadband DIA method, all ions generated within the source are passed through the quadrupole operating in rf-only mode and are allowed to collide with Ar within the collision cell of the instrument. The method was first described by Silva *et al.* for the quantitative analysis of complex proteomics samples, including an approach for the correlation of precursor and product ions using a chromatographic, in order to generate searchable product ion spectra, followed by description of the method by Plumb *et al.* for the qualitative analysis of small molecules/metabolites.^9,10^

To increase the selectivity of a broadband DIA (MS^E^) method, either IM separation or scanning of the quadrupole can be included into the acquisition schema. In ion mobility enabled DIA (HDMS^E^), fragmentation is induced after the IM separation in the Transfer T-Wave collision cell and, as such, fragment ions exhibit the same mobility characteristics as their precursor.^11^ This extra degree of specificity means that the use of a multidimensional acquisition system is required to track the mobility separation, which also dictates the use dedicated peak detection and deconvolution algorithms to acknowledge the multi-dimensional nature of the data. An addition to this method has been described where the collision energy is ramped as a function of drift time, instead of *m/z* and charge, providing improved coverage for more complex samples comparted to the originally proposed method.^12^

The scanning quadrupole DIA variant (SONAR) makes use of a scanning quadrupole and the same multidimensional acquisition mobility enabled DIA acquisition electronics employed. However, the first dimension now tracks the scanning quadrupole *m/z* value rather than the ion mobility separation, thereby providing enhanced fragment ion specificity, although at the loss in overall duty cycle.^13,14^ Typical resolving scanning windows are from 10-20 Da. The scan range is application dependent and normally from *m/z* 100-800 for small molecule/metabolite applications and from *m/z* 400-900 for peptide centric workflows. In principle, the generated ions exit the quadrupole at different times due to the scanning of the quadrupole and then undergo both low and higher energy collisions with Ar contained within the collision cell of the instrument. The oa-ToF analyzer acquires 200 mass spectra for each cycle of the quadrupole mass filter and the data acquired are stored in two separate data streams. Within the resolving isolation window, the peak profiles obtained from the low (MS1) and higher energy (MS2) data streams allows quadrupole filtered precursor ions, in addition to retention time, to be associated with the generated fragment ions.

SupplEmentary REFERENCES

(1) Pringle, S. D.; Giles, K.; Wildgoose, J. L.; Williams, J. P.; Slade, S. E.; Thalassinos, K.; Bateman, R. H.; Bowers, M. T.; Scrivens, J. H. An Investigation of the Mobility Separation of Some Peptide and Protein Ions Using a New Hybrid Quadrupole/Travelling Wave IMS/Oa-ToF Instrument. *Int. J. Mass Spectrom.* **2007**, *261*, 1–12.

(2) Giles, K.; Pringle, S. D.; Worthington, K. R.; Little, D.; Wildgoose, J. L.; Bateman, R. H. Applications of a Travelling Wave-Based Radio-Frequency-Only Stacked Ring Ion Guide. *Rapid Comm. Mass Spectrom.* **2004**, *18*, 2401–2414.

(3) Stahl, D. C.; Swiderek, K. M.; Davis, M. T.; Lee, T. D. Data-Controlled Automation of Liquid Chromatography/Tandem Mass Spectrometry Analysis of Peptide Mixtures. *J. Am. Soc. Mass 5pectrometry* **1996**, *7*, 532–540.

(4) McCormack, A. L.; Schieltz, D. M.; Goode, B.; Yang, S.; Barnes, G.; Drubin, D.; Yates, J. R. Direct Analysis and Identification of Proteins in Mixtures by LC/MS/MS and Database Searching at the Low-Femtomole Level. *Anal. Chem.* **1997**, *69*, 767–776.

(5) Bateman, R. H.; Carruthers, R.; Hoyes, J. B.; Jones, C.; Langridge, J. I.; Millar, A.; Vissers, J. P. C. A Novel Precursor Ion Discovery Method on a Hybrid Quadrupole Orthogonal Acceleration for Studying Protein Phosphorylation. *J. Am. Soc. Mass Spectrom.* **2002**, *13*, 792–803.

(6) Helm, D.; Vissers, J. P. C.; Hughes, C. J.; Hahne, H.; Ruprecht, B.; Pachl, F.; Grzyb, A.; Richardson, K.; Wildgoose, J.; Maier, S. K.; et al. Ion Mobility Tandem Mass Spectrometry Enhances Performance of Bottom-up Proteomics. *Mol. Cell. Proteomics* **2014**, *13*, 3709–3715.

(7) Mbasu, R. J.; Heaney, L. M.; Molloy, B. J.; Hughes, C. J.; Ng, L. L.; Vissers, J. P. C.; Langridge, J. I.; Jones, D. J. L. Advances in Quadrupole and Time-of-Flight Mass Spectrometry for Peptide MRM Based Translational Research Analysis. *Proteomics* **2016**, *16*, 2206–2220.

(8) Chen, Z.; Alelyunas, Y. W.; Wrona, M. D.; Kehler, J. R.; Szapacs, M. E.; Evans, C. A. Microflow UPLC and High-Resolution MS as a Sensitive and Robust Platform for Quantitation of Intact Peptide Hormones. *Bioanalysis* **2019**, *11*, 1275–1289.

(9) Silva, J. C.; Gorenstein, M. V.; Li, G.-Z.; Vissers, J. P. C.; Geromanos, S. J. Absolute Quantification of Proteins by LCMSE. *Mol. Cell. Biochem.* **2006**, *5*, 144–156.

(10) Plumb, R. S.; Johnson, K. A.; Rainville, P.; Smith, B. W.; Wilson, I. D.; Castro-Perez, J. M.; Nicholson, J. K. UPLC/MSE; a New Approach for Generating Molecular Fragment Information for Biomarker Structure Elucidation. *Rapid Commun. Mass Spectrom.* **2006**, *20*, 1989–1994.

(11) Rodriguez-Suarez, E.; Hughes, C.; Gethings, L.; Giles, K.; Wildgoose, J.; Stapels, M.; Fadgen, K. E.; Geromanos, S. J.; Vissers, J. P. C.; Elortza, F.; et al. An Ion Mobility Assisted Data Independent LC-MS Strategy for the Analysis of Complex Biological Samples. *Curr. Anal. Chem.* **2013**, *9*, 199–211.

(12) Distler, U.; Kuharev, J.; Navarro, P.; Levin, Y.; Schild, H.; Tenzer, S. Drift Time-Specific Collision Energies Enable Deep-Coverage Data-Independent Acquisition Proteomics. *Nat. Methods* **2014**, *11*, 167–170.

(13) Moseley, M. A.; Hughes, C. J.; Juvvadi, P. R.; Soderblom, E. J.; Lennon, S.; Perkins, S. R.; Thompson, J. W.; Steinbach, W. J.; Geromanos, S. J.; Wildgoose, J.; et al. Scanning Quadrupole Data-Independent Acquisition, Part A: Qualitative and Quantitative Characterization. *J. Proteome Res.* **2018**, *17*, 770–779.

(14) Gethings, L. A.; Richardson, K.; Wildgoose, J.; Lennon, S.; Jarvis, S.; Bevan, C. L.; Vissers, J. P. C.; Langridge, J. I. Lipid Profiling of Complex Biological Mixtures by Liquid Chromatography/Mass Spectrometry Using a Novel Scanning Quadrupole Data-Independent Acquisition Strategy. *Rapid Commun. Mass Spectrom.* **2017**, *31*, 1599–1606.

supplementary figure 1

1D extracted MS1 (left) and MS2 (right) data for a doubly charged analyte eluting at a retention time of 16.6 minutes within the peptide sample of tryptic peptides spiked into an *E. coli* matrix. The analyte ion is attributed to the DIVGAVLK peptide of yeast alcohol dehydrogenase. MS^E^ data can additionally be extracted in 2D, whilst HDMS^E^ and SONAR data can be further extracted in 3D.


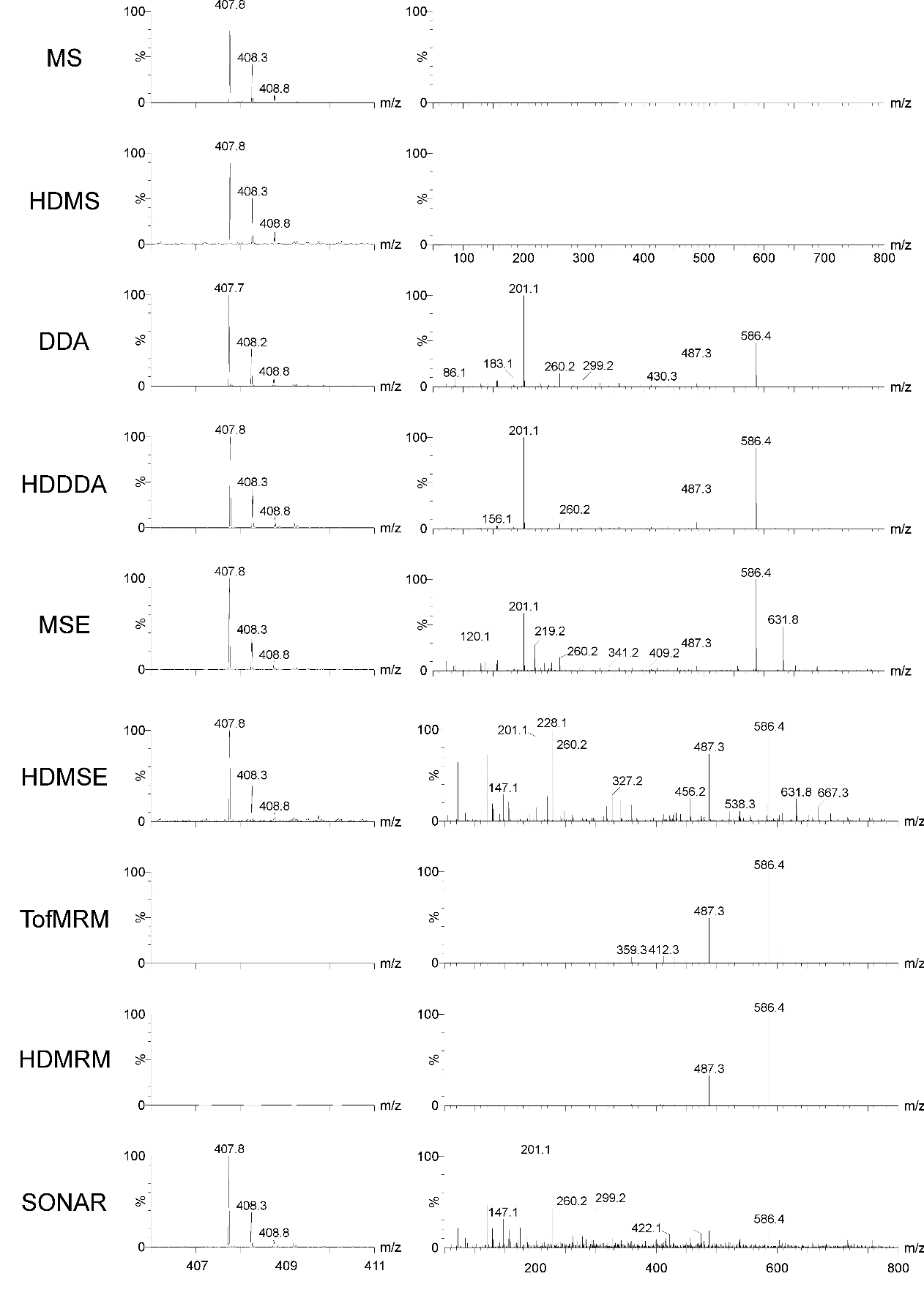


Supplementary figure 2

Comparison of uncorrected IM enabled DIA (HDMS^E^) data for analyte AKB-48 Apinaca 5-Hydroxypenytl with data corrected using the “isotope” and “MS2” methods. Blue dots = summed precursor signal (isotopes) uncorrected and corrected data points; blue line = sigmoidal fit; blue dashed lines = computational LLOQ and ULOQ estimates; orange line = linear fit.


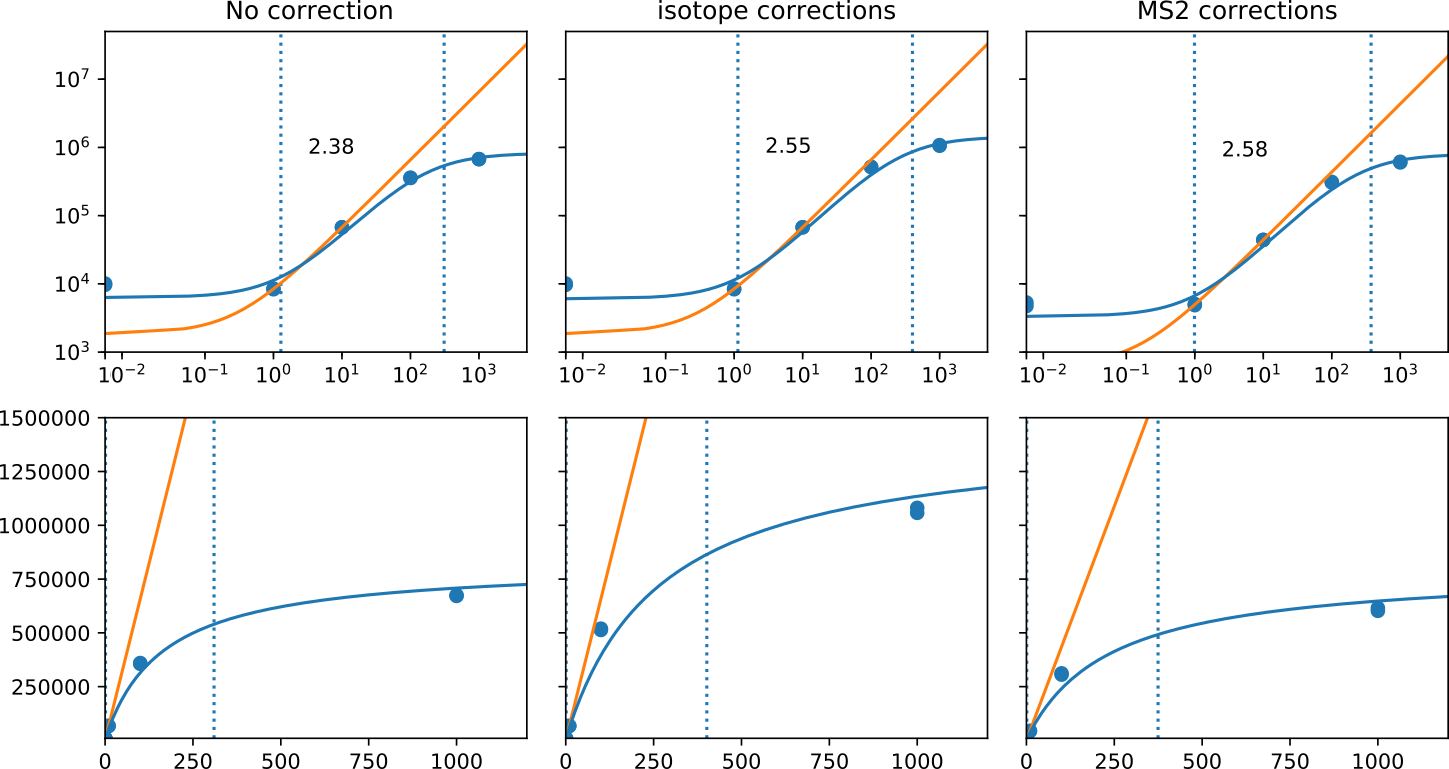


supplementary figure 3

Isotopic correction metabolites averaged out over all amounts injected on-column for IM-MS (HDMS) and IM enabled DIA (HDMS^E^) acquisition methods (left; orange = uncorrected; blue = corrected), the average gain in signal for the individual metabolite isotopes (top right; dark blue = IM-MS (HDMS); light blue = IM enabled DIA (HDMS^E^), and the overall signal gain summed over all isotopes and metabolites (bottom right; orange = uncorrected; blue = corrected).


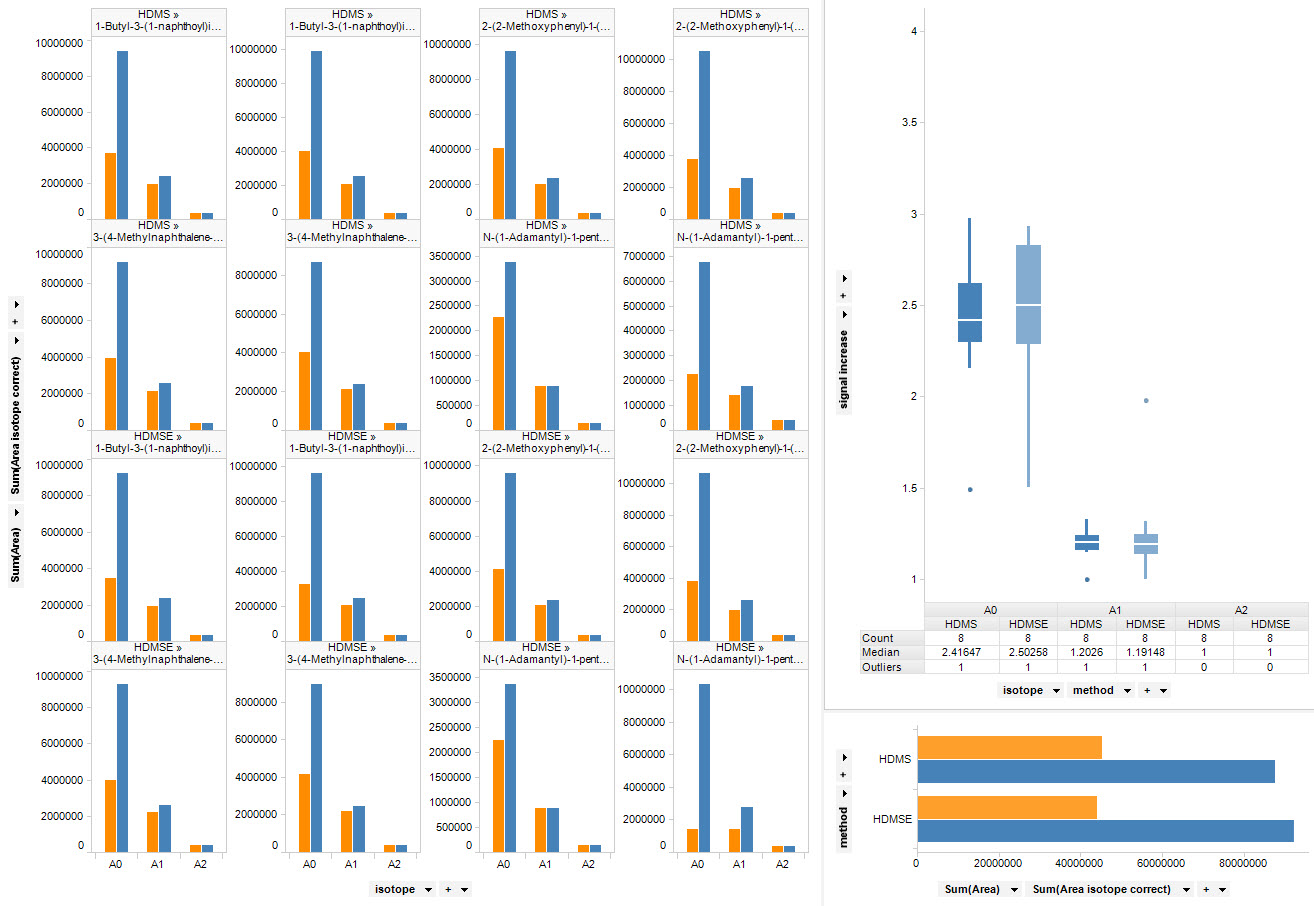


**SUPPLEMENTARY FIGURE 4**

Example corrected (using the best of the two correction methods) and uncorrected calibration curves for all acquisition methods are presented for peptide NLAENISR of rabbit Glycogen Phosphorylase B. Blue dots = measured response; blue lines = least square linear fit; blue dashed lines = computational LLOQ and ULOQ estimates; orange dashed lines = sigmoidal fit.


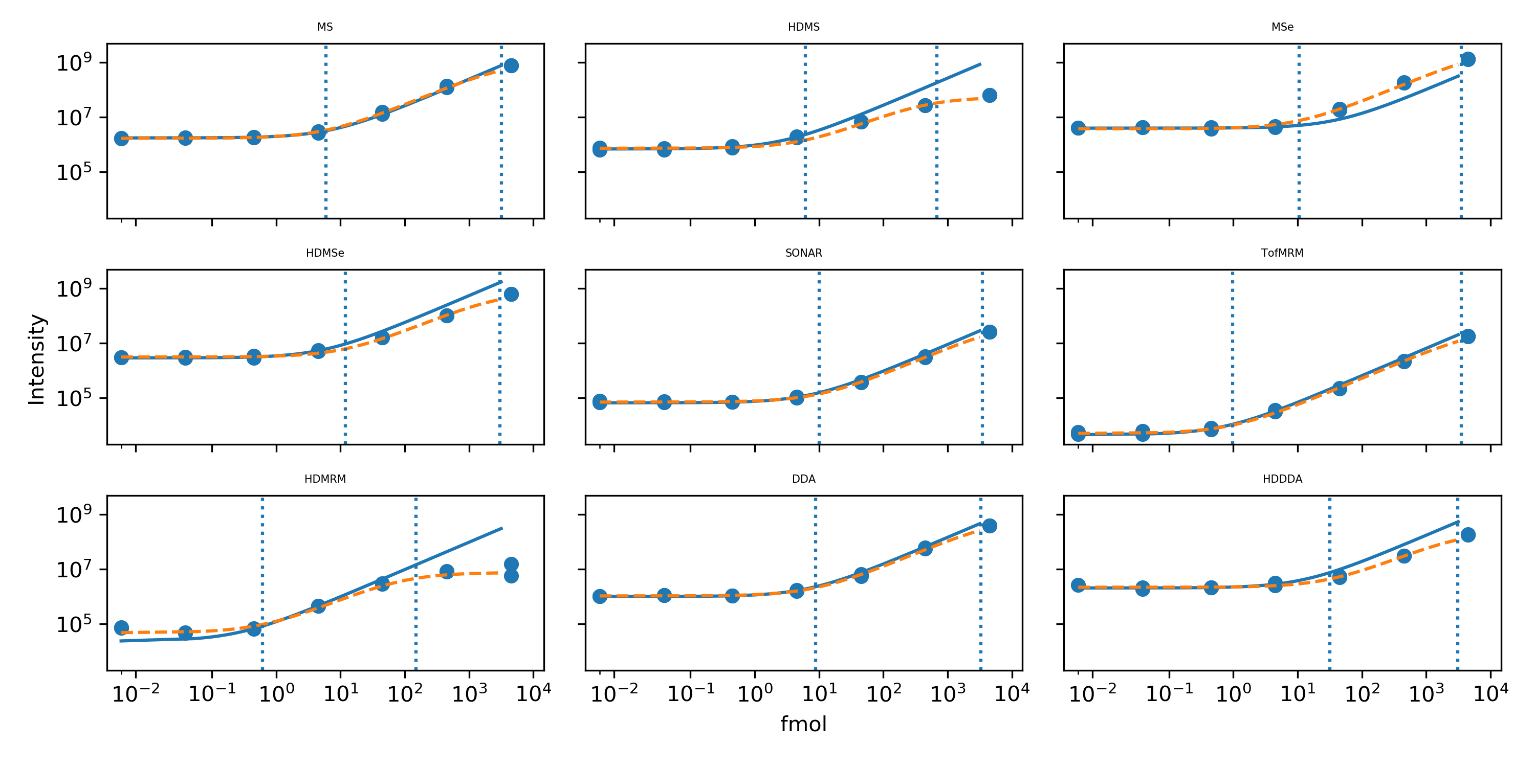


SupplEmentary table 1

The following MS acquisition parameters were used for metabolites throughout this study; -- = not applicable:

| **acquisition mode** | **acronym** | **Q1 isolation width (Da)** | **TWave velocity (m/s)** | **ToF resolution (FWHM)** | ***m/z* range** | **quadrupole range (Da)** | **acquisition time (s)** |
| --- | --- | --- | --- | --- | --- | --- | --- |
| MS | MS | -- | -- | 20,000 | 50 - 800 | -- | 0.2 |
| IM-MS | HDMS | -- | 1000 - 300 | 20,000 | 50 - 800 | -- | 0.2 |
| DDA | DDA | 2 | -- | 20,000 (MS1)  20,000 (MS2) | 50 - 800 | -- | 0.2  0.1 |
| DDA | HDDDA^†,∆^ | 2 | 2700 - 400 | 20,000 (MS1)  10,000 (MS2) | 50 - 5000 | -- | 0.2  0.1 |
| PRM | TofMRM | 2 | -- | 20,000 | 50 - 800 | -- | 0.15 |
| PRM | HDMRM^†^ | 2 | 2700 - 400 | 20,000 | 50 - 5000 | -- | 0.15 |
| broadband DIA | MS^E^ | -- | -- | 20,000 (MS1/MS2) | 50 - 800 | -- | 0.2 |
| IM enabled DIA | HDMS^E^ | -- | 1000 - 300 | 20,000 (MS1/MS2) | 50 - 800 | -- | 0.2 |
| scanning quadrupole DIA | SONAR | 10 | -- | 20,000 (MS1/MS2) | 50 - 850 | 100 - 800 | 0.2 |

^†^ Enhanced duty cycle (EDC), high duty cycle (HDC) or ion mobility (IM) enabled methods aimed at improving duty cycle (detail provided in Supplementary Note 1); ^∆^ Ion mobility (IM) was additionally used during the MS1 survey scan for charge state based precursor selection.

SupplEmentary table 2

The following MS acquisition parameters were used for (cross-linked) peptides throughout this study (-- = not applicable):

| **acquisition mode** | **acronym** | **Q1 isolation width (Da)** | **TWave velocity (m/s)** | **ToF resolution (FWHM)** | ***m/z* range** | **quadrupole range (Da)** | **acquisition time (s)** |
| --- | --- | --- | --- | --- | --- | --- | --- |
| MS | MS | -- | -- | 20,000 | 50 - 2000 | -- | 0.5 |
| IM-MS | HDMS | -- | 800 - 400 | 20,000 | 50 - 2000 | -- | 0.5 |
| DDA | DDA | 2 | -- | 20,000 (MS1)  20,000 (MS2) | 50 - 2000 | -- | 0.2  0.1 |
| DDA | HDDDA^†,∆^ | 2 | 2900 - 400 | 20,000 (MS1)  10,000 (MS2) | 50 - 2000 | -- | 0.2  0.1 |
| PRM | TofMRM | 2 | -- | 20,000 | 50 - 2000 | -- | 0.15 |
| PRM | HDMRM^†^ | 2 | -- | 20,000 | 50 - 2000 | -- | 0.25 |
| broadband DIA | MS^E^ | -- | -- | 20,000 (MS1/MS2) | 50 - 2000 | -- | 0.5 |
| IM enabled DIA | HDMS^E^ | -- | 800 - 400 | 20,000 (MS1/MS2) | 50 - 2000 | -- | 0.5 |
| scanning quadrupole DIA | SONAR | 20 | -- | 20,000 (MS1/MS2) | 50 - 2000 | 400 - 900 | 0.5 |

^†^ Enhanced duty cycle (EDC), high duty cycle (HDC) or ion mobility (IM) enabled methods aimed at improving duty cycle (detail provided in Methods Section and Supplementary Note 1); ^∆^ Ion mobility (IM) was additionally used during the MS1 survey scan for charge state based precursor selection.

SupplEmentary table 3

The following reference ^TW^CCS_N2_ values were used for metabolites throughout this study:

| **compound name** | **elemental composition** | **mw (g/mol)** | **^TW^CCS_N2_ (Å^2^)** |
| --- | --- | --- | --- |
| AKB-48 Apinaca 5-Hydroxypenytl metabolite | C_23_H_31_N_3_O_2_ | 381.5 | 201.5 |
| AKB-48 Apinaca 5-Hydroxypenytl metabolite-D_4_ | C_23_H_27_D_4_N_3_O_2_ | 385.5 | 201.4 |
| JWH-073 3-Hydroxybutyl metabolite | C_23_H_21_NO_2_ | 343.4 | 185.4 |
| JWH-073 3-Hydroxybutyl metabolite-D_5_ | C_23_H_16_D_5_NO_2_ | 348.5 | 185.3 |
| JWH-250 4-Hydroxypentyl metabolite | C_22_H_25_NO_3_ | 351.4 | 189.5 |
| JWH-250 4-Hydroxypentyl metabolite-D_5_ | C_23_H_20_D_5_NO_3_ | 356.5 | 189.4 |
| JWH-122 4-Hydroxypentyl metabolite | C_25_H_25_NO_2_ | 371.5 | 196.1 |
| JWH-122 4-Hydroxypentyl metabolite-D_5_ | C_25_H_20_D_5_NO_2_ | 376.5 | 196.0 |

SupplEmentary table 4

The semi-quantitative uncorrected figures of merit (average values summed over all peptides with acquisition, integration and/or computational outliers excluded from the analysis when passing a modified (Iglewicz and Hoaglin) z-score threshold; errors represent difference in analyte response/ionization efficiency) for TofMRM_EDC_ and TofMRM_sens_ modes of acquisition for peptides. Semi-quantitation is based on MS2 level.

**Peptides** (12 peptides; 7 concentration levels; duplicate injections)

| **acquisition mode** | **LLOQ (fmol)** | **# orders LDR** |
| --- | --- | --- |
| TofMRM_EDC_  TofMRM_sens_ | 0.2 ± 0.1  0.1 ± 0.0 | 4.1 ± 0.2  4.4 ± 0.1 |

SupplEmentary table 5

The following Skyline data files are available on Panorama (<https://panoramaweb.org/Age9Ya.url>) public repository:

| **sample name** | **analyte type** | **acquisition mode** |
| --- | --- | --- |
| DDA_2019-12-20_09-33-01.sky.zip | Metabolites | DDA |
| HDDDA_2019-12-20_09-34-19.zip | Metabolites | HDDDA |
| HDMRM_2019-12-20_09-35-12.zip | Metabolites | HDMRM |
| HDMS_2019-12-20_09-36-07.zip | Metabolites | HDMS |
| HDMSe_2019-12-20_09-37-57.zip | Metabolites | HDMS^E^ |
| MS_2019-12-20_09-39-32.zip | Metabolites | MS |
| MSe_2019-12-20_09-41-15.zip | Metabolites | MS^E^ |
| SONAR_2019-12-20_09-43-35.zip | Metabolites | SONAR |
| TofMRM_2019-12-20_09-46-47.zip | Metabolites | TofMRM |
| pDDA_2019-12-20_09-52-15.zip | Peptides | DDA |
| pHDDDA_2019-12-20_09-56-46.zip | Peptides | HDDDA |
| pHDMRM_2019-12-20_11-32-04.zip | Peptides | HDMRM |
| pHDMS_2019-12-20_11-39-15.zip | Peptides | HDMS |
| pHDMSE_2019-12-20_12-17-45.zip | Peptides | HDMS^E^ |
| pMS_2019-12-20_11-43-28.zip | Peptides | MS |
| pMSe_2019-12-20_11-53-00.zip | Peptides | MS^E^ |
| pSONAR_2019-12-20_12-03-01.zip | Peptides | SONAR |
| pTofMRM_2019-12-20_12-07-41.zip | Peptides | TofMRM |
| XLBSA_HDMSe_ASMS_2019-12-20_12-38-51.sky.zip | Cross-linked BSA | HDMS^E^ |
| XLBSA_MSe_ASMS_2019-12-20_12-41-58.sky.zip | Cross-linked BSA | MS^E^ |
| XLBSA_TofMRM_ASMS_2019-12-20_12-59-15.sky.zip | Cross-linked BSA | TofMRM |
